# Supplementary material for: Measuring and stimulating progress on implementing widely recommended food environment policies: the New Zealand case study
Source: Health Res Policy Syst. 2018 Jan 25;16:3. doi: 10.1186/s12961-018-0278-0 (PMC5785861; doi:10.1186/s12961-018-0278-0)
Supplement: Supplementary file 1 — Healthy Food Environment Policy Index (Food-EPI) tool and process. (DOCX 133 kb) [file 12961_2018_278_MOESM1_ESM.docx]

**Supplementary material 1**: *Healthy Food Environment Policy Index (Food-EPI) tool and process*

**

*SFigure 1 Components and domains of the Healthy Food Environment Policy Index (Food-EPI)*

Process driven by panel of independent and government public health experts

####

1.

Analyse context

2.

Collect relevant documents

3.

Evidence- ground the policies and actions

4.

Validate evidence with government officials

5.

Rate government policies and actions

6.

Identify and prioritize concrete actions

7.

Qualify, comment and recommend

8.

Translate results for government and stakeholders

*SFigure 2* *Process for assessing the extent of government policy implementation on food environments against international best practice using the Healthy Food Environment Policy Index (Food-EPI)*

***Stable 1*** *Importance and Achievability Criteria for prioritizing the actions identified by the expert panel*

| **Importance** | **Achievability** |
| --- | --- |
| Need  The size of the implementation gap | Feasibility  How easy or hard the action is to implement |
| Impact  The effectiveness of the action on improving food environments and diets (including reach and effect size) | Acceptability  The level of support from key stakeholders including government, the public, public health, and industry |
| Equity  Progressive / regressive effects on reducing food/diet-related health inequalities | Affordability  The cost of implementing the action |
| Other positive effects  (e.g., on protecting rights of children and consumers) | Efficiency  The cost-effectiveness of the action |
| Other negative effects  (e.g., regressive effects on household income, infringement of personal liberties) |  |
