# Supplementary material for: Measuring and stimulating progress on implementing widely recommended food environment policies: the New Zealand case study
Source: Health Res Policy Syst. 2018 Jan 25;16:3. doi: 10.1186/s12961-018-0278-0 (PMC5785861; doi:10.1186/s12961-018-0278-0)
Supplement: Supplementary file 2 — Healthy Food Environment Policy Index (Food-EPI) good practice indicators and recommendations made by the experts. (DOCX 62 kb) [file 12961_2018_278_MOESM2_ESM.docx]

***Supplementary material 2****: Healthy Food Environment Policy Index (Food-EPI) good practice indicators and recommendations made by the experts*

| GOOD PRACTICE INDICATOR | RECOMMENDATIONS |
| --- | --- |
| **FOOD COMPOSITION** | |
| **COMP1**: *Food composition targets/standards have been established by the government for the content of the nutrients of concern in certain foods or food groups if they are major contributors to population intakes of these nutrients of concern (trans fats and added sugars in processed foods, salt in bread, saturated fat in commercial frying fats)* | **COMP1V:** The Government strongly endorses existing and new sodium and sugar targets for the processed food groups that are major contributors to sodium and sugar intakes, consistent with international best practice targets  **COMP1M:** The Government sets mandatory sodium and sugar targets for the processed food groups that are major contributors to population sodium and sugar intakes, based on international best practice targets, and examines targets for saturated fat in processed foods  **Government agencies: MPI, FSANZ**  *Comment: All workshops agreed on the need for food composition targets but debated whether this recommendation should be mandatory or remain voluntary albeit with a much stronger role for Government. Therefore, there are 2 potential recommendations for this indicator. While sodium targets are well established internationally, sugar and saturated fat targets are less well established. In addition, for ‘added sugar’ to have targets, it would need to be declared on the nutrition information panel. Thus, the final recommendation focused on setting total sugar rather than added sugar targets for certain food groups.* |
| **COMP2**: *Food composition targets/standards have been established for out-of-home meals in food service outlets by the government for the content of the nutrients of concern in certain foods or food groups if they are major contributors to population intakes of these nutrients of concern (trans fats, added sugars, salt and saturated fat)* | **COMP2a:** The Government sets a mandatory standard for deep frying oils (maximum saturated & trans fats) for out-of-home meals & recommends targets for energy, sodium, saturated fat and sugar in Quick Service Restaurant (QSR) meals  **Government agencies: MPI, FSANZ**  *Comment: All workshops agreed on the need and feasibility to shift to mandatory standards for commercial deep frying fats and that recommended targets should be developed for QSRs for other nutrients of concern.* |
|  | **COMP2b:** The Government explores the incorporation of nutrition into the Food Safety requirements.  **Government agencies: MPI, FSANZ**  *Comment: Existing, robust systems to ensure food safety (including regulations, training, inspections, certification, surveys etc.) could be expanded to include the nutrition components under the system’s mandate to ‘protect public health’. This recommendation will require investigation on how to achieve this in practice.* |

| GOOD PRACTICE INDICATOR | RECOMMENDATIONS |
| --- | --- |
| **FOOD LABELLING** | |
| **LABEL1:** *Ingredient lists and nutrient declarations in line with Codex recommendations are present on the labels of all packaged foods* | **LABEL1a:** The Government requires added sugars to be added on the Nutrition Information Panel  **Government agencies: MPI, FSANZ**  *Comment: This issue is being explored already by FSANZ/MPI in New Zealand and the US FDA already requires added sugars to be on the Nutrition Facts Panel. Three out of four of the workshops considered that the rationale, evidence and feasibility were already compelling enough for added sugars to be required in the Nutrition Information Panel.* |
|  | **LABEL1b:** The Government requires the types of fats that are used to be added in the ingredient list  **Government agencies: MPI, FSANZ**  *Comment: The participants at all workshops agreed that the type of fat (e.g. palm oil, sunflower oil) should be required in the ingredient list rather than a generic term (e.g. vegetable oil).* |
|  | **LABEL1c:** The Government requires trans fats to be added in the Nutrition Information Panel where they exceed a particular level  **Government agencies: MPI, FSANZ**  *Comment: While the overall trans fat intake in New Zealand is below the WHO recommended intake level, some of the workshops considered it was important to include this safeguard since some specific products may have relatively high levels of trans fats.* |

| GOOD PRACTICE INDICATOR | RECOMMENDATIONS |
| --- | --- |
| **FOOD LABELLING** | |
| **LABEL2:** *Robust, evidence-based regulatory systems are in place for approving/reviewing claims on foods, so that consumers are protected against unsubstantiated and misleading nutrition and health claims* | **LABEL2:** The Government investigates the application of the Nutrient Profiling Scoring Criterion (NPSC) to restrict the use of nutrient content claims on packaged unhealthy foods (especially ‘irrelevant claims’ such as ‘no cholesterol’ claims on plant-based foods)  **Government agencies: MPI, FSANZ**  *Comment: The participants at all workshops agreed the presence of nutrition content claims (especially irrelevant claims) on unhealthy foods was misleading and NZ’s world-benchmark restrictions for health claims should be extended to nutrition content claims.* |
| **LABEL3:** *A single, consistent, interpretive, evidence-informed front-of-pack supplementary nutrition information system, which readily allows consumers to assess a product’s healthiness, is applied to all packaged foods* | **LABEL3:** The Government urgently addresses anomalies in the design of the Health Star Ratings, including the algorithm (especially for sugar), increases promotion, and makes the HSR mandatory if not widespread uptake by 2019.  **Government agencies: MPI, FSANZ, Health Promotion Agency**  *Comment: All workshops agreed on the need for urgent action to improve the performance and integrity of the HSR. Some participants said that the “increasing promotion” should only occur after the anomalies which are damaging the credibility of the HSR have been addressed. The lack of uptake by the food industry was the rationale for the making the HSR mandatory.* |
| **LABEL4:** *A consistent, single, simple, clearly-visible system of labelling the menu boards of all quick service restaurants (i.e. fast food chains) is applied by the government, which allows consumers to interpret the nutrient quality and energy content of foods and meals on sale* | **LABEL4a:** The Government requires all Quick Service Restaurants (QSR) to display kJ labelling on their menu boards  **LABEL4b:** The Government investigates KJ labelling on menu boards in independent fast food outlets  **Government agencies: MPI, FSANZ**  *Comment: All workshops agreed that implementing kJ labelling for QSRs, as is occurring in Australia, should go ahead in New Zealand but one workshop also had an additional recommendation to investigate KJ labelling on menu boards in independent fast food outlets.* |

| GOOD PRACTICE INDICATOR | RECOMMENDATIONS |
| --- | --- |
| **FOOD PROMOTION** | |
| **PROMO1:** *Effective policies are implemented by the government to restrict exposure and power of promotion of unhealthy foods to children through broadcast media (TV, radio)* | **PROMO1:** The Government introduces regulations to restrict unhealthy food marketing, as defined by the WHO nutrient profiling model, to children up to 18 years through broadcast media, including during children's peak viewing times (e.g. evening period up to 9pm), and investigates the incorporation/nutrient profiling of brands and companies in the restriction of unhealthy food marketing  **Government agencies: MoH**  *Comment: All workshops agreed on the need for government regulations given the lack of evidence of effectiveness of the self-regulatory approaches that New Zealand uses. Since advertising of brands or companies only (i.e. no specific food advertised) is an obvious loophole which companies will exploit participants felt that the nutrient profiling of brands and companies needed to be explored to close that loophole.* |
| **PROMO2:** *Effective policies are implemented by the government to restrict exposure and power of promotion of unhealthy foods to children through non-broadcast media (e.g. Internet, social media, food packaging, sponsorship, outdoor advertising including around schools)* | **PROMO2:** The Government introduces regulations to restrict unhealthy food marketing, as defined by the WHO nutrient profiling model, to children up to 18 years through non- broadcast media, including food packaging, sport sponsorship and social media, and investigates the incorporation/nutrient profiling of brands and companies in the restriction of unhealthy food marketing.  **Government agencies: MoH**  *Comment: All workshops agreed on this recommendation. The investigation of nutrient profiling of brands and companies was similarly added as for recommendation PROMO1.* |
| **PROMO3:** *Effective policies are implemented by the government to ensure that unhealthy foods are not commercially promoted to children in settings where children gather (e.g. preschools, schools, sport and cultural events)* | **PROMO3:** The Government introduces regulations to restrict unhealthy food marketing, as defined by the WHO nutrient profiling model, in children's settings (covering children up to 18 years), and investigates the incorporation/nutrient profiling of brands and companies in the restriction of unhealthy food marketing.  **Government agencies: MoH**  *Comment: All workshops agreed on this recommendation. The investigation of nutrient profiling of brands and companies was similarly added as for recommendation PROMO1.* |

| GOOD PRACTICE INDICATOR | RECOMMENDATIONS |
| --- | --- |
| **FOOD PRICES** | |
| **PRICES1:** *Taxes on healthy foods are minimised to encourage healthy food choices where possible (e.g. low or no sales tax, excise, value-added or import duties on fruit and vegetables)* | **PRICES1:** The Government removes GST from unprocessed fruits and vegetables  **Government agencies: Treasury, MoH**  *Comment: The workshops were divided on this recommendation (2 proposed this recommendation and 2 proposed no recommendation) because of different assessments of the costs and benefits and the equity impacts of such a policy.* |
| **PRICES2:** *Taxes on unhealthy foods (e.g. sugar-sweetened beverages, foods high in nutrients of concern) are in place to discourage unhealthy food choices where possible, and these taxes are reinvested to improve population health* | **PRICES2:** The Government introduces a 20% tax on sugar-sweetened beverages and explores use of revenue for programs to improve public health and/or wellbeing.  **Government agencies: Treasury, MoH**  *Comment: All workshops agreed on this recommendation.* |
| **PRICES3:** *The intent of existing subsidies on foods, including infrastructure funding support (e.g. research and development, supporting markets or transport systems), is to favour healthy rather than unhealthy foods* | **PRICES3:** The Government requires government programs that subsidize/supply food for children to meet food and nutrition guidelines  **Government agencies: MoH, MoE**  *Comment: All workshops agreed on this recommendation, although only a few such programs exist in New Zealand.* |
| **PRICES4:** *The government ensures that food-related income support programs are for healthy foods* | **PRICES4:** The Government explores subsidies for low income people for healthy foods and ensures emergency benefits includes healthy foods  **Government agencies: MoH, Treasury**  *Comment: Most workshops proposed no recommendation since such subsidies were minor in New Zealand, but one workshop proposed this exploratory action.* |

| GOOD PRACTICE INDICATOR | RECOMMENDATIONS |
| --- | --- |
| **FOOD PROVISION** | |
| **PROV1:** *The government ensures that there are clear, consistent policies (including nutrition standards) implemented in schools and early childhood education services for food service activities (canteens, food at events, fundraising, promotions, vending machines etc.) to provide and promote healthy food choices* | **PROV1:** The Government enacts policies that ensure schools and early childhood education services to provide/sell foods that meet the MOH food and beverage classification system (updated in March 2016)  **Government agencies: MoE, MoH**  *Comment: All workshops agreed on this recommendation* |
| **PROV2:** *The government ensures that there are clear, consistent policies in other public sector settings for food service activities (canteens, food at events, fundraising, promotions, vending machines, public procurement standards etc.) to provide and promote healthy food choices* | **PROV2:** The Government makes the developed healthy food and drink policy (including the green/amber/red food classification system) mandatory throughout the Government health sector and recommends the policy for other public settings  **Government agencies: MoH, DHBs**  *Comment: All workshops agreed on this recommendation which builds on the momentum within the government health sector, converting voluntary approaches to mandatory for that sector, and advocating for voluntary uptake in other government sectors.* |
| **PROV3:** *The government ensures that there are good support and training systems to help schools and other public sector organisations and their caterers meet the healthy food service policies and guidelines* | **PROV3:** The Government increases funding for support and training of Government and children’s settings to remove barriers and stimulate implementation of policies and actions to create healthy food environments  **Government agencies: MoH, MoE, MPI**  *Comment: All workshops agreed on this recommendation and particularly reducing the barriers for implementation (e.g. excessive food safety requirements which favour unhealthy, packaged foods and prevent fresh foods being offered).* |
| **PROV4:** *Government actively encourages and supports private companies to provide and promote healthy foods and meals in their workplaces* | **PROV4:** The Government increases funding for support and training for private sector settings and organizations to develop policies and actions to create healthy food environments.  **Government agencies: MoH**  *Comment: All workshops agreed on this recommendation.* |

| GOOD PRACTICE INDICATOR | RECOMMENDATIONS |
| --- | --- |
| **FOOD RETAIL** | |
| **RETAIL1:** *Zoning laws and policies are robust enough and are being used, where needed, by local governments to place limits on the density or placement of quick serve restaurants or other outlets selling mainly unhealthy foods in communities* | **RETAIL1:** The Government enacts legislation to allow local Government to create healthy community food environments for children (e.g. school zones)  **Government agencies: MoH, MPI**  *Comment: All workshops agreed on this recommendation recognising the need for legislation to strengthen zoning laws to permit local government and community action around schools.* |
| **RETAIL2:** *Zoning laws and policies are robust enough and are being used, where needed, by local governments to encourage the availability of outlets selling fresh fruit and vegetables.* | **RETAIL2:** The Government investigates the options for removing the barriers and restrictions for outlets selling fresh fruit and vegetables  **Government agencies: MoH, MPI**  *Comment: Most workshops agreed on this recommendation to investigate options for increasing the availability of fresh, healthy foods.* |
| **RETAIL3:** *The Government ensures existing support systems are in place to encourage food stores to promote the in-store availability of healthy foods and to limit the in-store availability of unhealthy foods* | **RETAIL3:** The Government supports the food industry to develop SMART (Specific, Measurable, Achievable, Relevant, Time Bound) pledges as part of the Healthy Kids Industry Pledge and evaluates those pledges.  **Government agencies: MoH**  *Comment: All workshops agreed on this recommendation with debate about the descriptor SMART versus SMARTER (includes evaluate, redesign). The latter implies that companies already have SMART pledges whereas, the vast majority of current pledges are non-specific and unmeasurable.* |
| **RETAIL4:** *The government ensures existing support systems are in place to encourage food service outlets to increase the promotion and availability of healthy foods and to decrease the promotion and availability of unhealthy foods* | **RETAIL4:** The Government engages with and supports the food service industry to phase out unhealthy food practices (e.g. refill cups, large portion sizes).  **Government agencies: MoH**  *Comment: Most workshops considered this recommendation to be one of government engaging meaningfully with the food service industry to identify and support the voluntary phasing out of industry practices which are promoting unhealthy diets.* |

| GOOD PRACTICE INDICATOR | RECOMMENDATIONS |
| --- | --- |
| **FOOD TRADE** | |
| **TRADE1:** *The direct and indirect impacts of international trade and investment agreements on food environments and population nutrition and health are assessed and considered* | **TRADE1:** The Government includes formal and explicit population nutrition and health risk assessments as part of their national interest analysis on trade and investment agreements  **Government agencies: MoH, MFAT**  *Comment: All workshops agreed on this recommendation.* |
| **TRADE2:** *The government adopts measures to manage investment and protect their regulatory capacity with respect to public health nutrition* | **TRADE2:** The Government ensures that specific and explicit provisions are included in trade and investment agreements, allowing the New Zealand government to preserve its regulatory capacity to protect and promote public health  **Government agencies: MoH, MFAT**  *Comment: All workshops agreed on this recommendation.* |

| GOOD PRACTICE INDICATOR | RECOMMENDATIONS |
| --- | --- |
| **LEADERSHIP** | |
| **LEAD1:** *There is strong, visible, political support (at the Head of State / Cabinet level) for improving food environments, population nutrition, diet-related NCDs and their related inequalities* | **LEAD1:** The Government sets a target to reduce childhood overweight and obesity by 8 percentage-points (from one-third to one-quarter) by 2025 with decreasing inequalities  **Government agencies: Cabinet, Minister of Health, MoH**  *Comment: All workshops agreed on the need for a prevalence and inequalities target. The precise size of the target was debated, with the final proposal being to achieve Australia’s current prevalence rates of childhood overweight/obesity (one in four) by 2025 with decreasing inequalities.* |
| **LEAD2:** *Clear population intake targets have been established by the government for the nutrients of concern to meet WHO and national recommended dietary intake levels.* | **LEAD2:** The Government sets clear targets for the reduction of population salt, sugar and saturated fat intake based on WHO recommendations.  ***Government agencies: Cabinet, Minister of Health MoH***  *Comment: All workshops agreed on this recommendation.* |
| **LEAD3:** *Clear, interpretive, evidence-informed food-based dietary guidelines have been established and implemented.* | **LEAD3:** The Government actively implements and increases funding to promote the new Eating and Activity guidelines fully and translates them into the social, environmental and cultural context.  **Government agencies: Minister of Health, MoH**  *Comment:* All workshops agreed on the need for actively implementing and promoting all aspects of the new guidelines but successive workshops noted that these need to be translated for the socio-cultural and education contexts and for environmental sustainability. |
| **LEAD4:** *There is a comprehensive, transparent, up-to-date implementation plan (including priority policy and program strategies, social marketing for public awareness and threat of legislation for voluntary approaches) linked to national needs and priorities, to improve food environments, reduce the intake of the nutrients of concern to meet WHO and national recommended dietary intake levels, and reduce diet-related NCDs* | **LEAD4a:** The Government revises and strengthens the childhood and adolescent obesity plan through including more actions focused on creating healthy food environments and increases funding for the implementation & evaluation of plan  **Government agencies: Cabinet, Minister of Health, MoH**  *All workshops agreed on this recommendation.* |
|  | **LEAD4b:** The Government develops, funds and implements a comprehensive national nutrition action plan to prevent dietary related NCDs in NZ  **Government agencies: Cabinet, Minister of Health, MoH**  *Comment: Some of the workshops proposed this recommendation in addition to the revised childhood obesity plan since they felt there was the need to focus on nutrition more broadly.* |
| **LEAD5:** *Government priorities have been established to reduce inequalities in relation to diet, nutrition, obesity and NCDs.* | **LEAD5a:** The Government includes specific policy objectives and targets within the childhood obesity plan to more directly reduce health inequities and inequalities.  **Government agencies: Cabinet, Minister of Health, MoH**  *Comment: All workshops felt that the childhood obesity plan was an important place to focus on inequalities through both targeted programs and pro-equity national policies.* |
|  | **LEAD5b:** The Government includes specific objectives and targets within the national nutrition plan to more directly reduce health inequities and inequalities  **Government agencies: Cabinet, Minister of Health, MoH**  *Comment: Those workshops which proposed a national nutrition plan included this recommendation because of the very high nutritional inequalities in New Zealand.* |

| GOOD PRACTICE INDICATOR | RECOMMENDATIONS |
| --- | --- |
| **GOVERNANCE** | |
| **GOVER1:** *There are robust procedures to restrict commercial influences on the development of policies related to food environments where they have conflicts of interest with improving population nutrition* | **GOVER1:** The Government strengthens the conflict of interest procedures to ensure that food industry representatives with direct conflicts are not included in setting food-related policy objectives and principles (this does not apply to their participation in policy implementation).  **Government agencies: Minister of Health, MoH, Sate Services Commission**  *Comment: All workshops agreed on this recommendation.* |
| **GOVER2:** *Policies and procedures are implemented for using evidence in the development of food policies* | **GOVER2:** The Science Advisors to Ministers who are engaged in policy development related to food and nutrition should work with a government-appointed nutrition scientific committee to ensure that policies are evidence based.  **Government agencies: Minister of Health, MoH**  *Comment: The Science Advisors were seen as important positions to strengthen the inclusion of evidence in policy-making but workshops felt that there still needed to be an external nutrition advisory committee to support the assessment of nutrition evidence for decision-making.* |
| **GOVER3:** *Policies and procedures are implemented for ensuring transparency in the development of food policies* | **GOVER3:** The Government creates a government lobby register to require detailed reporting on lobbying and introduces public declaration of political donations.  **Government agencies: Cabinet, State Services Commission**  *Comment: All workshops agreed that this is an important recommendation for reducing conflicts of interest.* |
| **GOVER4:** *The government ensures access to comprehensive nutrition information and key documents (e.g. budget documents, annual performance reviews and health indicators) for the public* | No recommendation made |

| GOOD PRACTICE INDICATOR | RECOMMENDATIONS |
| --- | --- |
| **MONITORING** | |
| **MONIT1:** *Monitoring systems, implemented by the government, are in place to regularly monitor food environments (especially for food composition for nutrients of concern, food promotion to children, and nutritional quality of food in schools and other public sector settings), against codes/guidelines/standards/targets* | **MONIT1:** The Government regularly monitors food environments with focus on food composition, food marketing, food in schools and public sector settings and the price of healthy versus unhealthy foods using Consumer Price Index data  **Government agencies: MoH, MPI, Statistics NZ**  *Comment: All workshops agreed on this recommendation.* |
| **MONIT2:** *There is regular monitoring of adult and childhood nutrition status and population intakes against specified intake targets or recommended daily intake levels* | **MONIT2:** The Government conducts a new national nutrition survey for children to be organized in the next 3 years.  **Government agencies: Minister of Health, MoH, MPI**  *Comment: A new national nutrition survey for children was considered the most important recommendation for this indicator by all workshops. The last one was conducted in 2002 and the size of the nutritional problems in childhood and adolescence warranted up-to-date data on their dietary intake and nutritional status. A new adult nutrition survey should also be planned for and a mechanism for regular nutrition surveys for the future*. |
| **MONIT3:** *There is regular monitoring of adult and childhood overweight and obesity prevalence using anthropometric measurements.* | **MONIT3:** The Government develops a system to deliver regular fine-grained estimates of overweight and obesity prevalence (especially for children and adolescents) e.g. in-School check in addition to B4School checks.  **Government agencies: Minister of Health, Minister of Education, MoH, MoE**  *Comment: All workshops agreed on this recommendation, noting the value of the universal coverage for B4School Check and the absence of such systems for school-aged children.* |
| **MONIT4:** *There is regular monitoring of adult and childhood overweight and obesity prevalence using anthropometric measurements* | **MONIT4:** The Government continues to invest in cardiovascular disease and diabetes risk assessments and investigates the inclusion of height and weight measurements and the use of the data for population monitoring.  **Government agencies: Minister of Health, MoH**  *Comment: All workshops agreed on this recommendation.* |
| **MONIT5:** *There is sufficient evaluation of major programs and policies to assess effectiveness and contribution to achieving the goals of the nutrition and health plans* | **MONIT5:** The Government includes robust process and impact programme evaluations in any major investment made to improve population nutrition.  **Government agencies: Minister of Health, MoH**  *Comment: All workshops agreed on this recommendation.* |
| **MONIT6:** *Progress towards reducing health inequalities and societal and economic determinants of health are regularly monitored* | **MONIT6:** The Government funds regular monitoring reports on the underlying societal and economic determinants of health and the related progress on the reduction of health inequalities.  **Government agencies: Cabinet, Minister of Health, MoH**  *Comment: All workshops agreed on this recommendation*. |

| GOOD PRACTICE INDICATOR | RECOMMENDATIONS |
| --- | --- |
| **FUNDING** | |
| **FUND1:** *The ‘Population Nutrition Promotion’ budget, as a proportion of total health spending and/or in relation to the diet-related NCD burden is sufficient to reduce diet-related NCDs. Section to be updated after all OIAs received* | **FUND1:** The Government increases funding for population nutrition promotion to at least 10% of obesity/overweight health care costs.  **Government agencies: Minister of Finance, Treasury, Minister of Health, MoH**  *Comment: All workshops agreed on the need to increase funding for prevention of obesity and population nutrition promotion and felt that a benchmark for the amount could be about 10% of the direct health care costs of overweight and obesity (which are probably now close to $1billion annually).* |
| **FUND2:** Government funded research is targeted for improving food environments, reducing obesity, NCDs and their related inequalities | **FUND2:** The Government ensures that improving nutrition and reducing nutrition inequalities is a priority funding stream within the Science Challenges.  **Government agencies: MBIE**  *Comment: All workshops recognised the need for nutrition priorities in research and that the National Sciences Challenges were an appropriate mechanism to ensure this. The High Value Nutrition NSC was mentioned as one which could have better and more equitable population nutrition as an objective.* |
| **FUND3:** *There is a statutory health promotion agency in place that includes an objective to improve population nutrition, with a secure funding stream* | **FUND3:** The level of Health Promotion Agency funding allows to focus on high profile hard-hitting social marketing campaigns on healthy eating.  **Government agencies: Health Promotion Agency, Minister of Health**  *Comment: All workshops agreed on this recommendation to lift nutrition literacy in New Zealand.* |

| GOOD PRACTICE INDICATOR | RECOMMENDATIONS |
| --- | --- |
| **PLATFORMS** | |
| **PLATF1:** *There are robust coordination mechanisms across departments and levels of government (national and local)) to ensure policy coherence, alignment, and integration of food, obesity and diet-related NCD prevention policies across governments* | **PLATF1:** The Government strengthens and expands platforms for engagement for food-related prevention policies across Government (national and local).  **Government agencies: Minister of Health, MoH, MPI**  *Comment: All workshops agreed on this recommendation to strengthen national platforms for engagement but also noted the need for New Zealand to also strongly engage with and honour international commitments such as to Sustainable Development Goals, the WHO NCD action plan, and the WHO implementation plan of the Commission on Ending Childhood obesity.* |
| **PLATF2:** *There are formal platforms between government and the commercial food sector to implement healthy food policies* | **PLATF2:** The Government strengthens the engagement platform around the industry pledges as part of the Healthy Kids Industry Pledge.  **Government agencies: Minister of Health, MoH, MPI**  *Comment: All workshops agreed on this recommendation.* |
| **PLATF3:** *There are formal platforms for regular interactions between government and civil society on food policies and other strategies to improve population nutrition* | **PLATF3:** The Government ensures formal platforms with civil society, including a nutrition advisory committee  **Government agencies: Minister of Health, MoH**  *Comment: All workshops agreed on this recommendation.* |
| **PLATF4:** *The government leads a broad, effective and sustainable systems-based approach with local organisations to improve the healthiness of food environments at a national level* | **PLATF4:** The Government takes lessons from Healthy Families New Zealand and other regional systems platforms to expand systems approaches and to create more sustainable systems platforms.  **Government agencies: Minister of Health, MoH, DHBs**  *Comment: All workshops agreed on this recommendation.* |

| GOOD PRACTICE INDICATOR | RECOMMENDATIONS |
| --- | --- |
| **HEALTH IN ALL POLICIES** | |
| **HIAP1:** *There are processes in place to ensure that population nutrition, health outcomes and reducing health inequalities are considered and prioritised in the development of all government policies relating to food* | **HIAP1:** MPI and MBIE assess the wider health impact of food policies (not only from a safety point of view) on long-term population health, to ensure that food policies are compatible with the objectives of improving population nutrition and reducing obesity and diet-related NCDs and their inequalities.  **Government agencies: Minister of Health, Minister for MBIE, MPI, MBIE, MoH**  *Comment: All workshops agreed on this recommendation.* |
| **HIAP2:** *There are processes (e.g. health impact assessments) to assess and consider health impacts during the development of other non-food policies* | **HIAP2:** The Government establishes a health impact assessment (HIA) capacity, including funding for HIAs at the national and local level, to ensure that government policies in general are compatible with the objectives of improving health.  **Government agencies: Minister of Health, MoH**  *Comment: All workshops agreed on this recommendation.* |

**Abbreviations**

DHB: District Health Board; FSANZ: Food Standards Australia New Zealand; HIA: Health Impact Assessment; HSR: Health Star Ratings; MBIE: Ministry of Business, Innovation and Employment; MFAT: Ministry of Foreign Affairs and Trade; MOE: Ministry of Education; MPI: Ministry for Primary Industries; NCD: Non-communicable diseases; NPSC: Nutrient Profiling Scoring Criterion; QSR: Quick Service Restaurants; WHO: World Health Organization
